# Supplementary material for: Amino acid sensor GCN2 promotes SARS-CoV-2 receptor ACE2 expression in response to amino acid deprivation
Source: Commun Biol. 2022 Jul 1;5:651. doi: 10.1038/s42003-022-03609-0 (PMC9249868; doi:10.1038/s42003-022-03609-0)
Supplement: Supplementary file 2 — Supplementary Information [file 42003_2022_3609_MOESM2_ESM.pdf]

Supplementary information

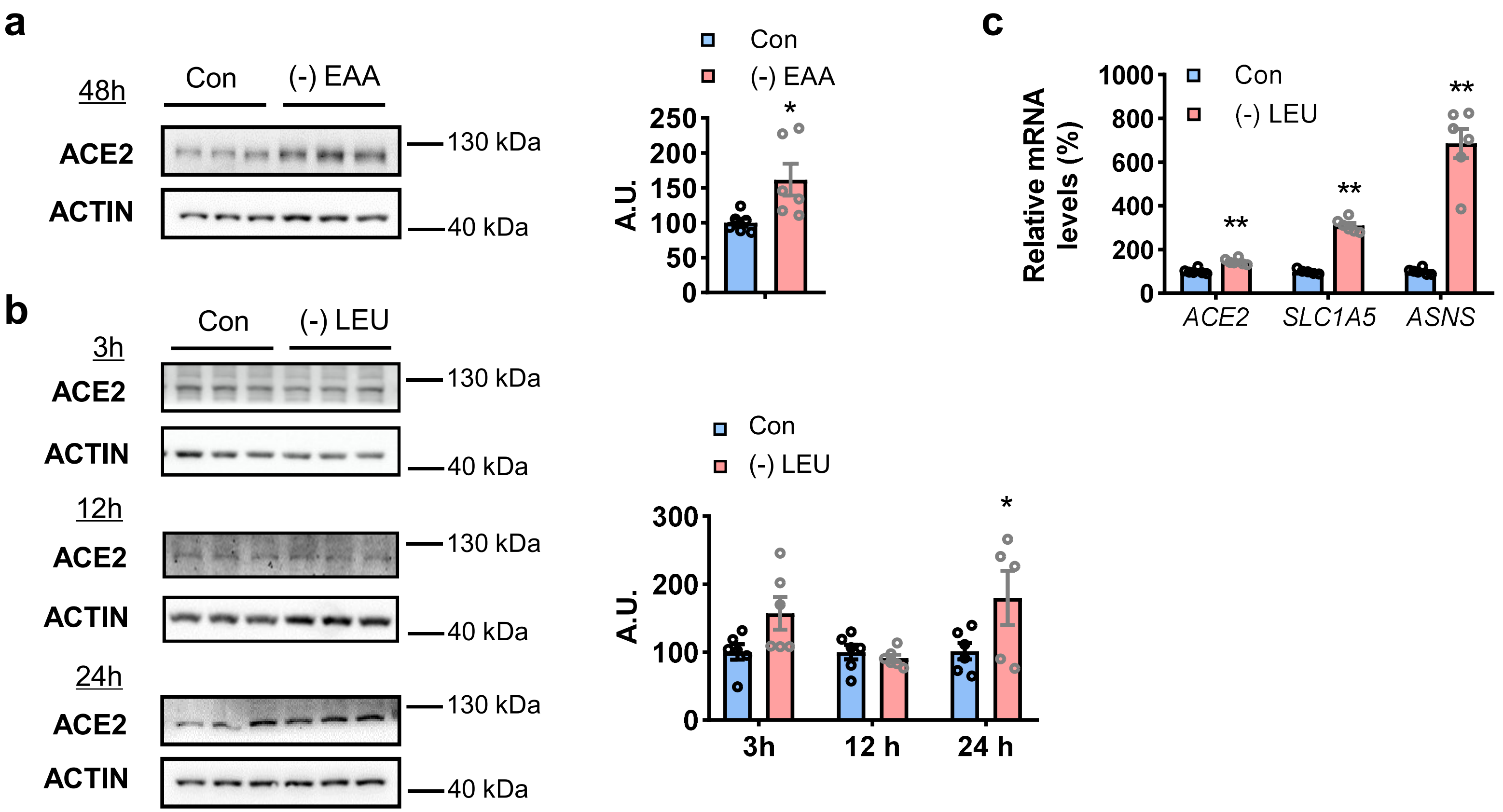

Supplementary Figure 1. ACE2 expression is upregulated response to amino acid deprivation.
(a) CCD 841 cells were incubated with control (Con) or essential amino acid starvation ((-) EAA) medium for 48 h. ACE2 protein levels were analyzed by western blotting (left) and quantified by densitometric analysis (right); A.U.: arbitrary unit. (b) CCD 841 cells were incubated with Con or leucine starvation ((-) LEU) medium for 3 h, 12 h or 24 h, respectively. ACE2 protein levels were analyzed by western blotting (left) and quantified by densitometric analysis (right); A.U.: arbitrary unit. (c) CCD 841 cells were incubated with Con or leucine deprivation ((-) LEU) medium for 48 h. The mRNAs of *ACE2*, *SLC1A5*, and *ASNS* were analyzed by qRT-PCR. Data are expressed as the mean $\pm$ SEM (n = 5–6 per group, as indicated by scatter circles). * $P < 0.05$, ** $P < 0.01$.

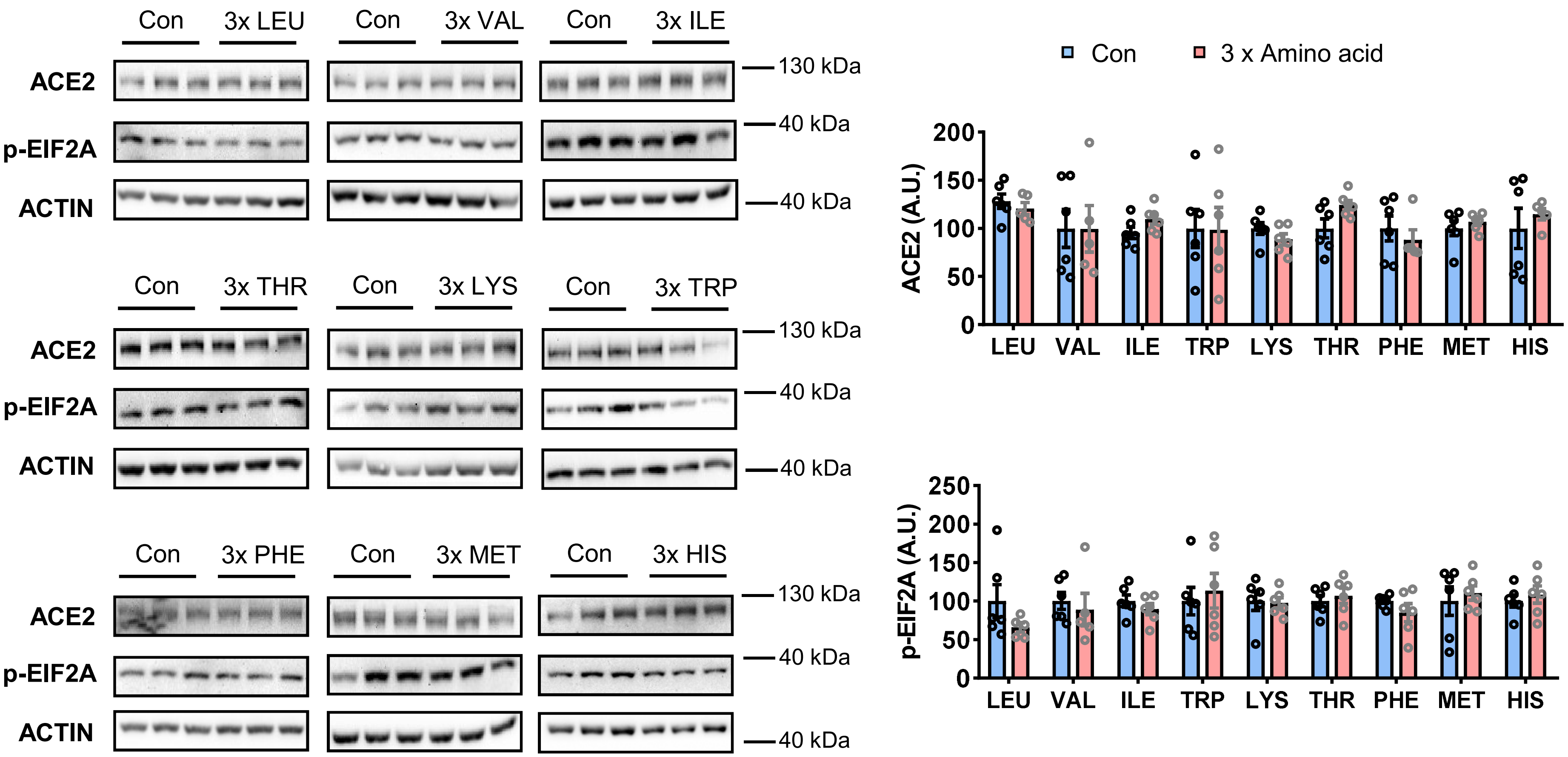

Supplementary Figure 2. Three-fold essential amino acid incubation has no effect on ACE2 expression.
CCD 841 cells were incubated with control culture medium (Con) or 3-fold essential amino acid (leucine, LEU; Valine, VAL; Isoleucine, ILE; Threonine, THR; Lysine, LYS; Tryptophan, TRP; Phenylalanine, PHE; Methionine, MET; Histidine, HIS) culture medium for 48 h, respectively. ACE2 and p-EIF2A expression were analyzed by western blotting (left) and quantified by densitometric analysis (right); A.U.: arbitrary unit. Data are expressed as the mean ± SEM (n = 5–6 per group, as indicated by scatter circles).

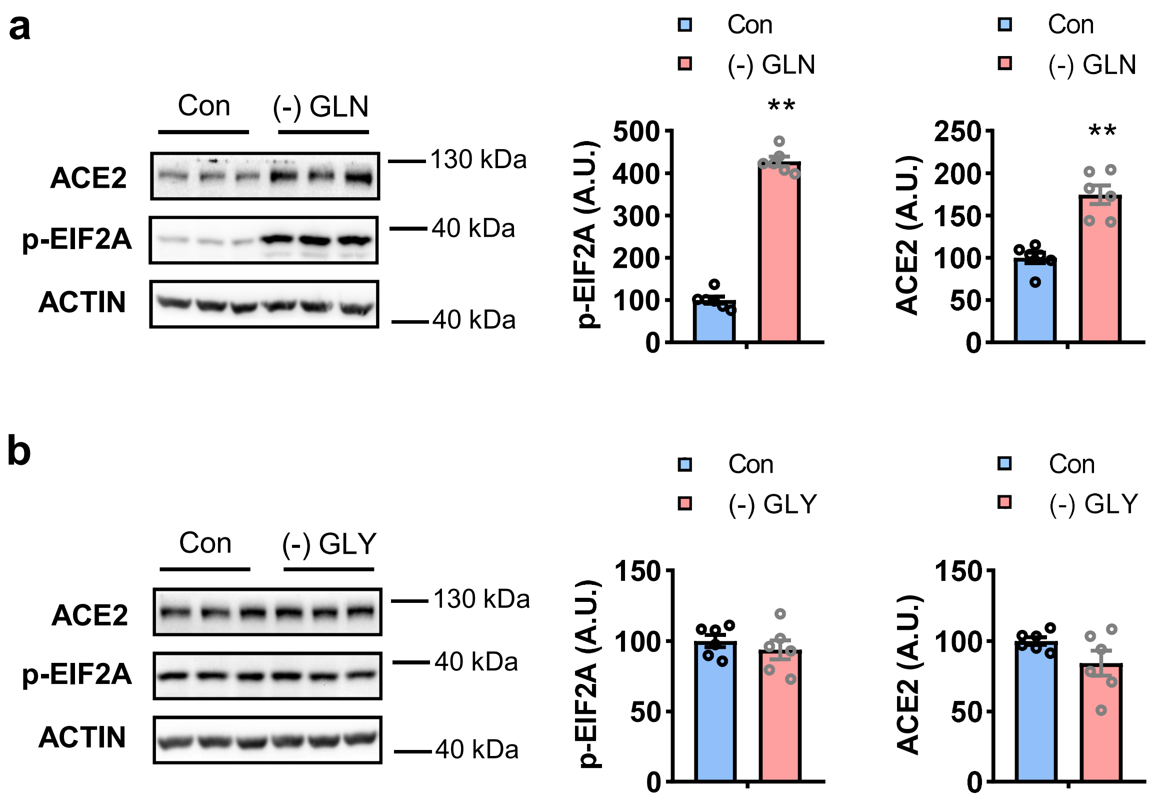

Supplementary Figure 3. The effects of non-essential amino acid starvation on ACE2 expression.
(a) and (b) CCD 841 cells were incubated with control (Con), glutamine starvation ((-)GLN), or glycine starvation ((-)GLY) culture medium for 48 h, respectively. ACE2 and p-EIF2A expression were analyzed by western blotting (left) and quantified by densitometric analysis (right); A.U.: arbitrary unit. Data are expressed as the mean ± SEM (n = 5–6 per group, as indicated by scatter circles). ** *P* < 0.01.

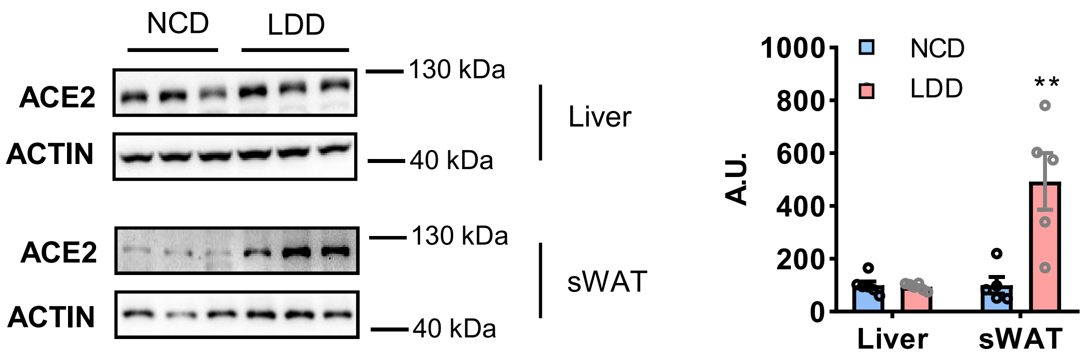

Supplementary Figure 4. The ACE2 protein expression in liver and subcutaneous white adipose tissue (sWAT) under leucine deprivation. 10-week-old male wild type mice were fed with control diet (NCD) or leucine deprivation diet (LDD) for 7 days. ACE2 protein levels in liver and sWAT were analyzed by western blotting (left) and quantified by densitometric analysis (right); A.U.: arbitrary unit. Data are expressed as the mean ± SEM (n = 5–6 mice per group, as indicated by scatter circles). * $P < 0.05$, ** $P < 0.05$.

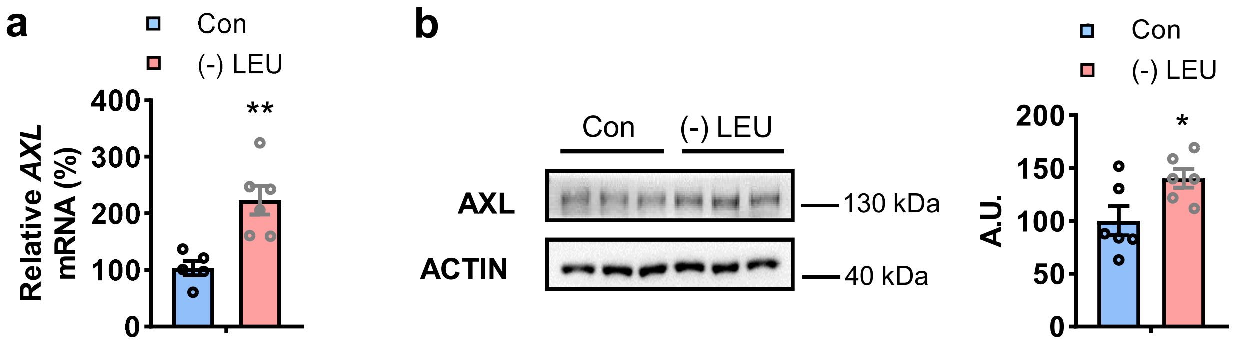

Supplementary Figure 5. Leucine deprivation increased tyrosine-protein kinase receptor UFO (AXL) expression.
(a) and (b) CCD 841 cells were incubated with control culture medium (Con) or leucine starvation ((-) LEU) culture medium for 48 h. (a) mRNA expression of *AXL* was analyzed by qRT-PCR. (b) AXL protein levels were analyzed by western blotting (left) and quantified by densitometric analysis (right); A.U.: arbitrary unit. Data are expressed as the mean ± SEM (n = 6 per group, as indicated by scatter circles). * $P < 0.05$, ** $P < 0.01$.

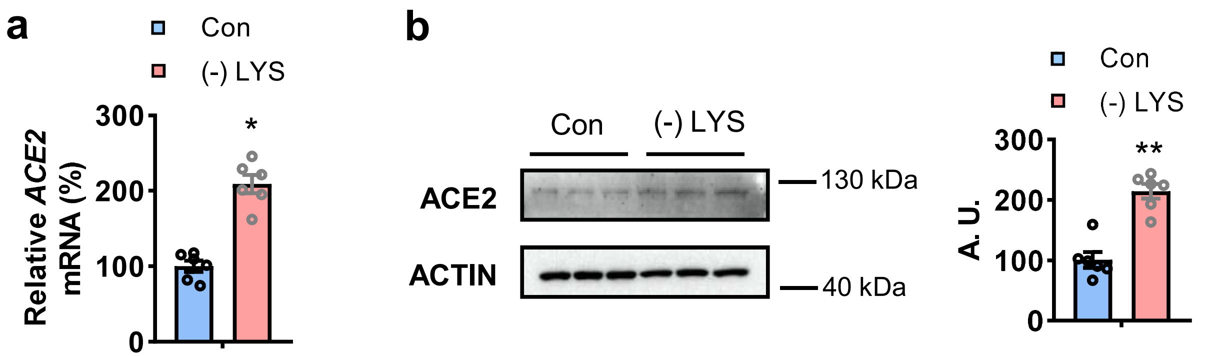

Supplementary Figure 6. Lysine deprivation increased ACE2 expression in human bronchial epithelial BEAS-2B cells.
(a) and (b) BEAS-2B cells were incubated with control culture medium (Con) or lysine starvation ((-) LYS) culture medium for 48 h. (a) mRNA expression of *ACE2* was analyzed by qRT-PCR. (b) ACE2 protein levels were analyzed by western blotting (left) and quantified by densitometric analysis (right); A.U.: arbitrary unit. Data are expressed as the mean ± SEM (n = 6 per group, as indicated by scatter circles). * $P < 0.05$, ** $P < 0.01$.

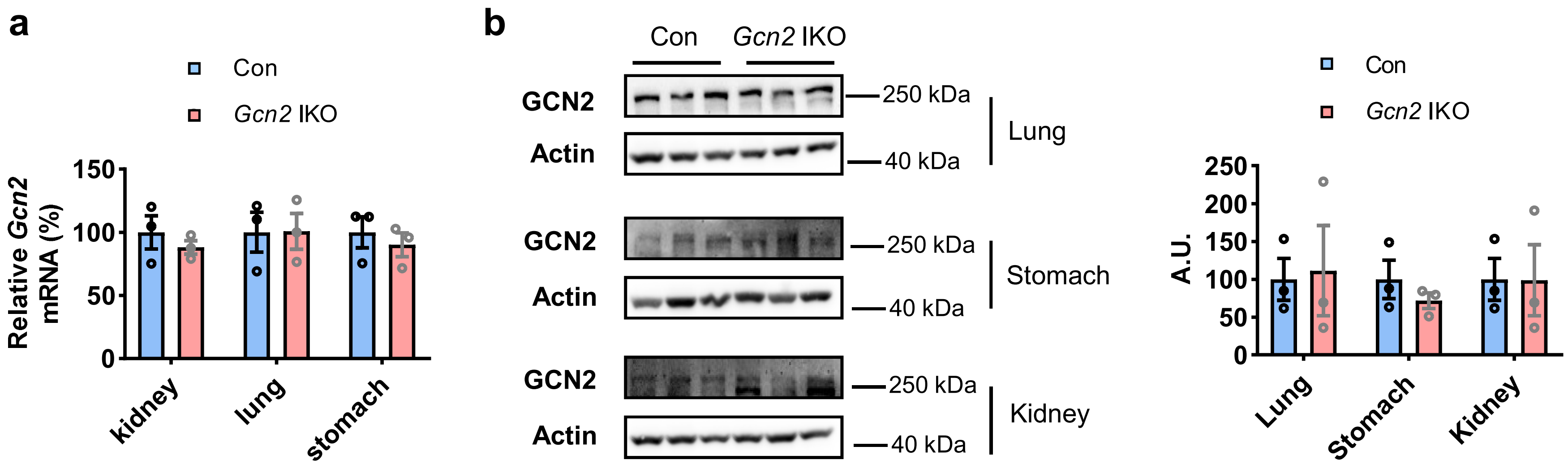

Supplementary Figure 7. GCN2 expression in lung, stomach, and kidney of intestinal epithelial cell-specific *Gcn2* deletion (*Gcn2* IKO) mice. (a) *Gcn2* mRNA levels were analyzed by RT-PCR. Studies were conducted using 10-week-old male *Gcn2*-floxed (Con) or *Gcn2* IKO mice. Data are expressed as the mean ± SEM (n = 3 mice per group). (b) GCN2 protein levels in lung, stomach, and kidney were analyzed by western blotting (left) and quantified by densitometric analysis (right); A.U.: arbitrary unit. Studies were conducted using 10-week-old male *Gcn2*-floxed (Con) or *Gcn2* IKO mice. Data are expressed as the mean ± SEM (n = 3 mice per group, as indicated by scatter circles).

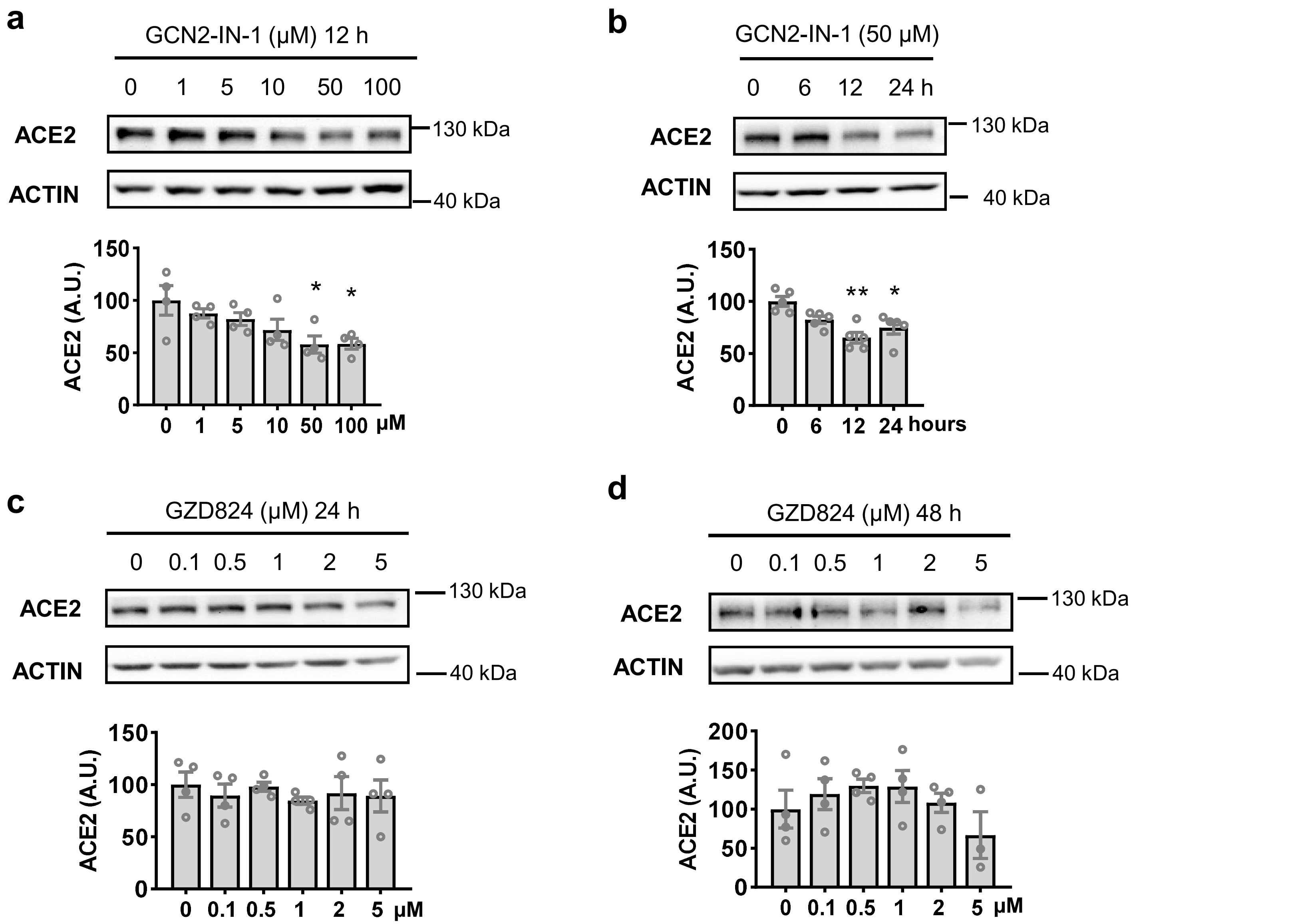

Supplementary Figure 8. GCN2 inhibitors reduced ACE2 expression in *vitro*.
(a–d) CCD841 cells were incubated with indicated concentration of GCN2 inhibitors, GCN2-IN-1 (A-92, HY-100877) and GZD824 (HY-15666), for indicated time. ACE2 protein levels were analyzed by western blotting (left) and quantified by densitometric analysis (right); A.U.: arbitrary unit. Data are expressed as the mean ± SEM (n = 4–6 per group, as indicated by scatter circles). * $P < 0.05$, ** $P < 0.01$.

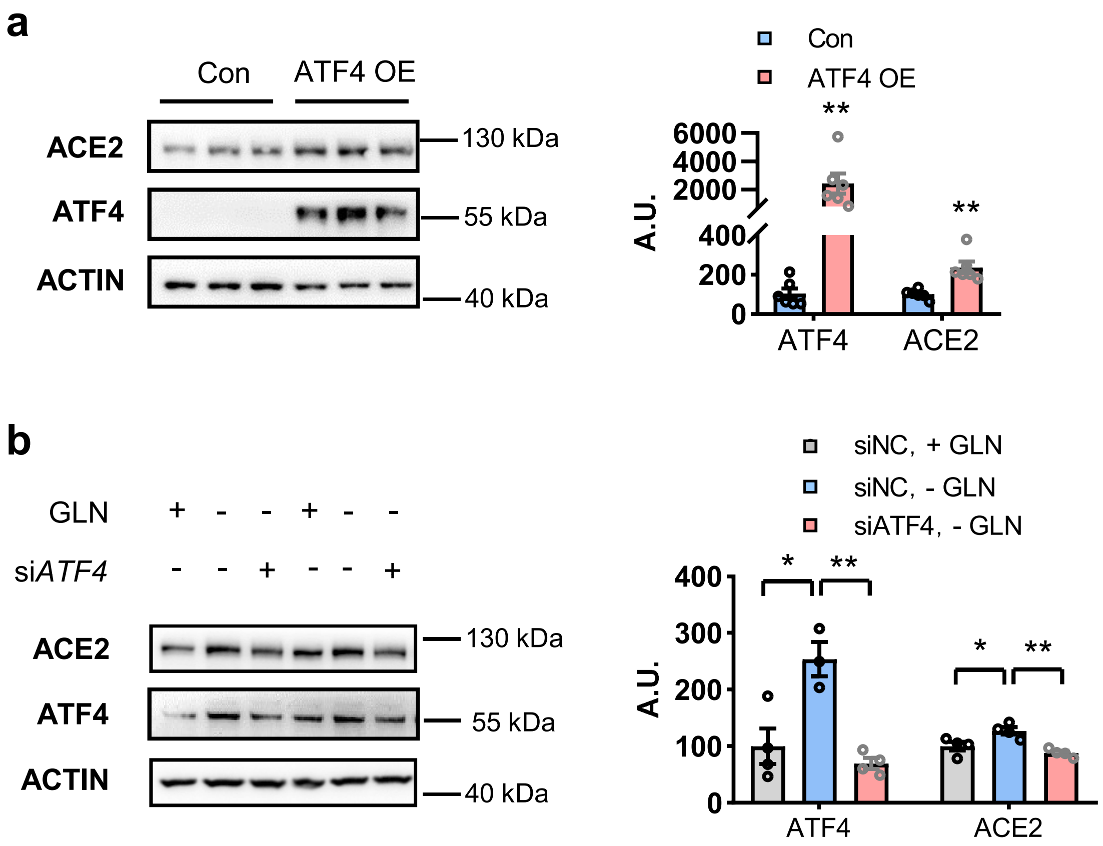

Supplementary Figure 9. ATF4 regulates ACE2 expression under amino acid deprivation in *vitro*.
(a) CCD841 cells were transfected with control plasmid (Con) or plasmid expressing human ATF4 (ATF4 OE) for 48 h. (b) CCD841 cells were transfected with control small interfering RNAs (*siNC*) or small interfering RNAs targeting at human ATF4 (*siATF4*). After 12 h, cells were incubated with either control (+ GLN) or glutamine starvation (- GLN) culture medium for 48 h. ATF4 and ACE2 protein levels were analyzed by western blotting (left) and quantified by densitometric analysis (right); A.U.: arbitrary unit. Data are expressed as the mean $\pm$ SEM (n = 3–6 per group, as indicated by scatter circles). * $P < 0.05$; ** $P < 0.01$.

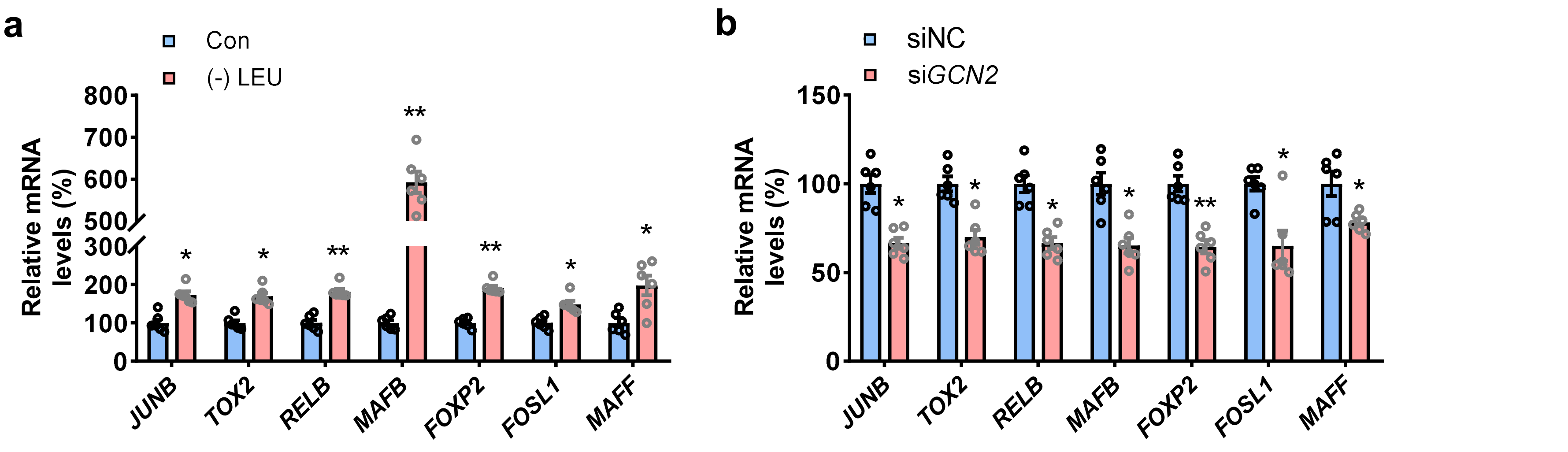

Supplementary Figure 10. Validating the mRNA expression of differential transcription factors from RNA-seq data.
(a) CCD 841 cells were incubated with control culture medium (Con) or leucine starvation ((-) LEU) culture medium for 48 h. (b) CCD841 cells were transfected with control small interfering RNAs (*siNC*) or small interfering RNAs targeting at human *GCN2* (*siGCN2*) for 48 h. mRNA expression of *MAFB*, *MAFF*, *FOSL1*, *JUNB*, *RELB*, *TOX2*, and *FOXP2* were analyzed by qRT-PCR. Data are expressed as the mean ± SEM (n = 6 per group, as indicated by scatter circles). * $P < 0.05$, ** $P < 0.01$.

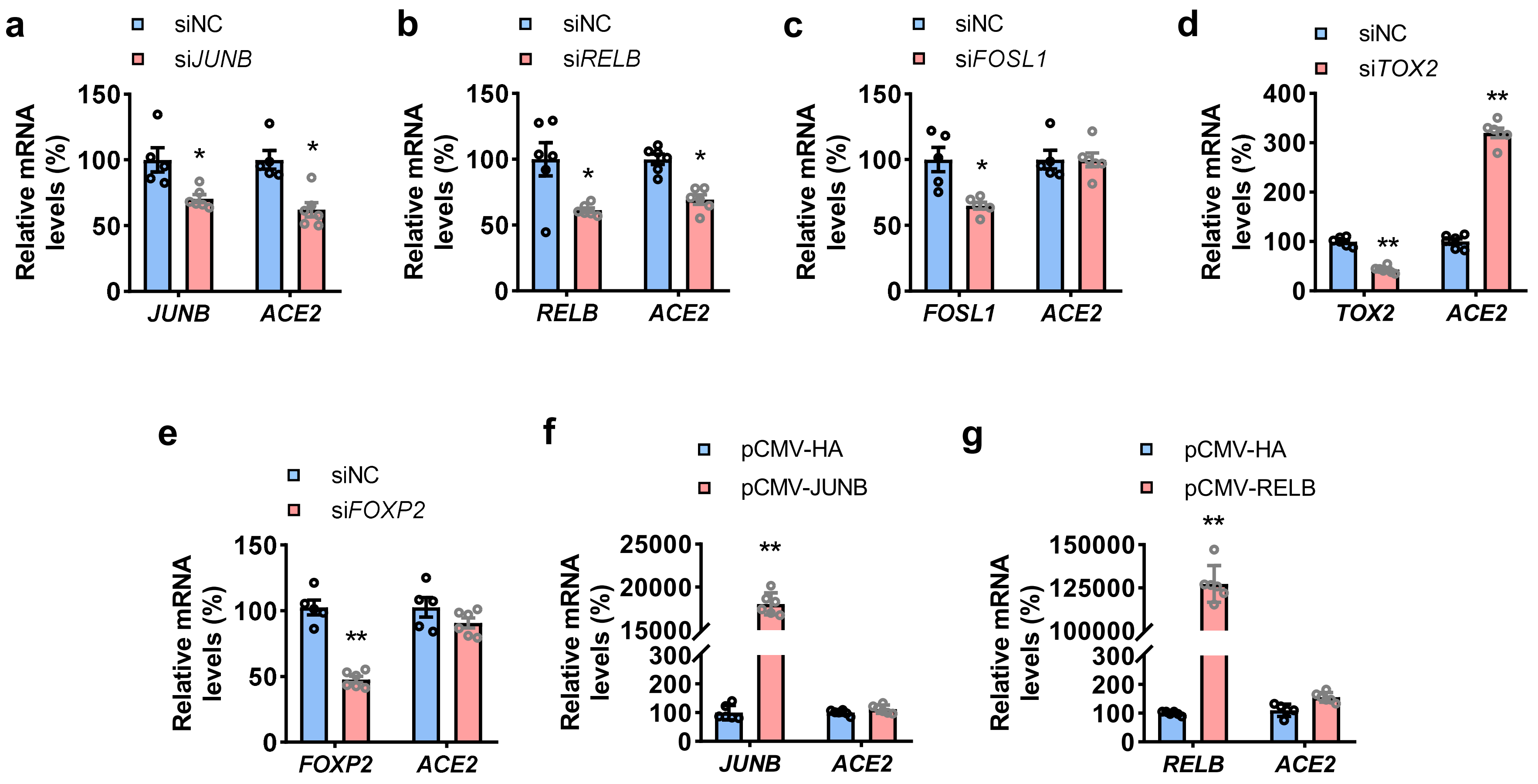

Supplementary Figure 11. Other transcription factors have no effect on ACE2 expression.
(a–e) CCD841 cells were transfected with control small interfering RNAs (*siNC*) or small interfering RNAs targeting at human *FOSL1* (*siFOSL1*), *JUNB* (*siJUNB*), *RELB* (*siRELB*), *TOX2* (*siTOX2*), or *FOXP2* (*siFOXP2*) for 48 h. mRNA expression of *ACE2*, *FOSL1*, *JUNB*, *RELB*, *TOX2*, and *FOXP2* were analyzed by qRT-PCR. (f-g) CCD841 cells were transfected with control plasmid (pCMV-HA) or plasmids expressing human *JUNB* (pCMV-*JUNB*), or *RELB* (pCMV-*RELB*) for 48 h. mRNA expression of *ACE2*, *JUNB*, and *RELB* were analyzed by qRT-PCR. Data are expressed as the mean ± SEM (n = 6 per group, as indicated by scatter circles). * $P < 0.05$, ** $P < 0.01$.

Figure 1a

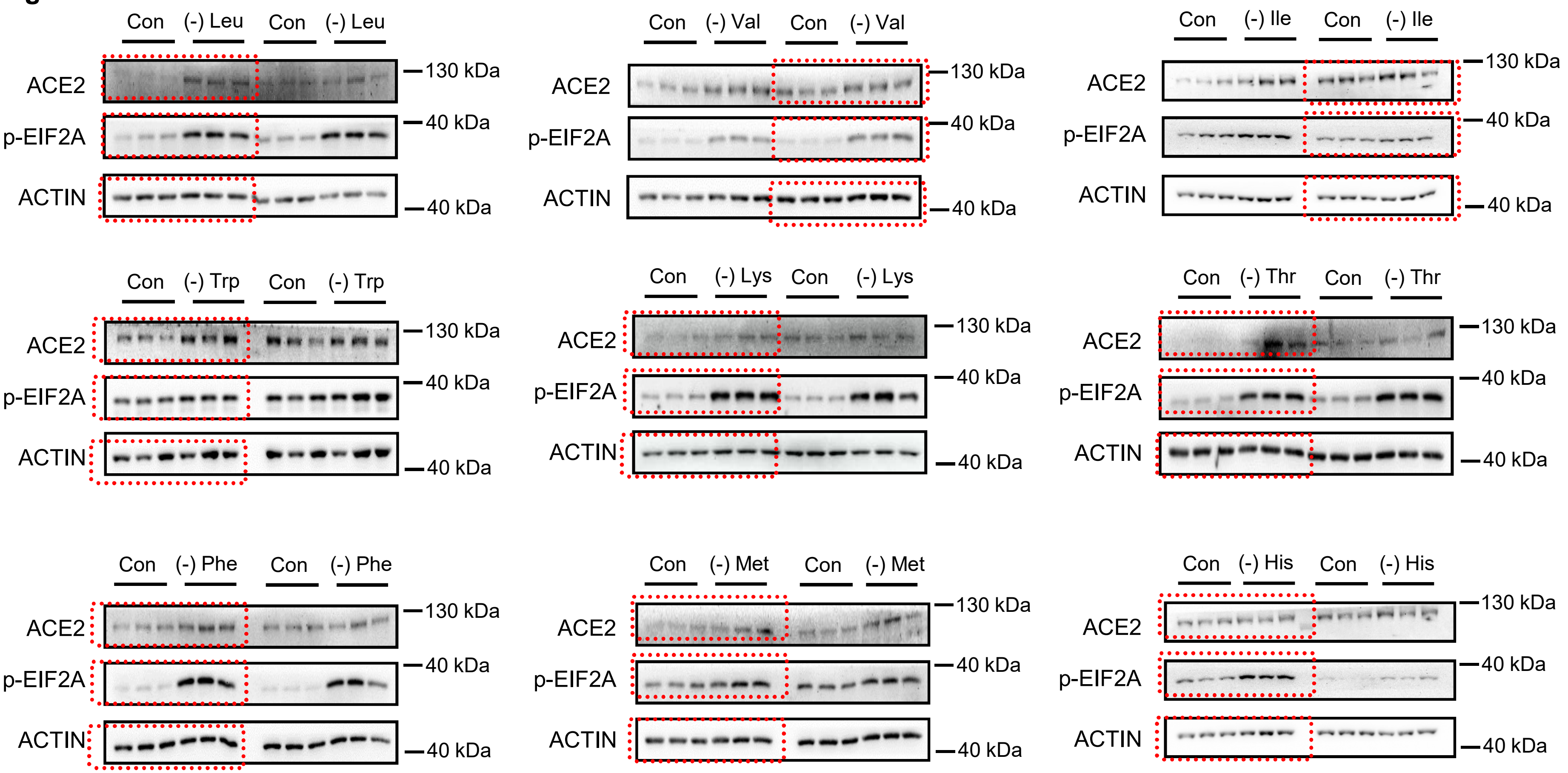

Figure 1c

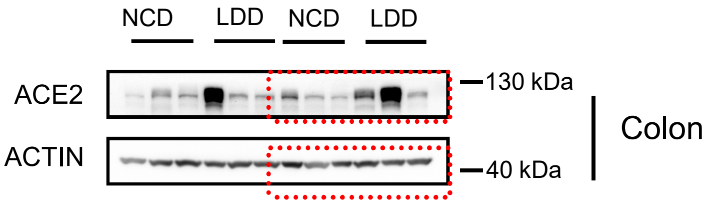

Supplementary Figure 12. Unedited blot for Figure 1a and 1c.

Figure 2b

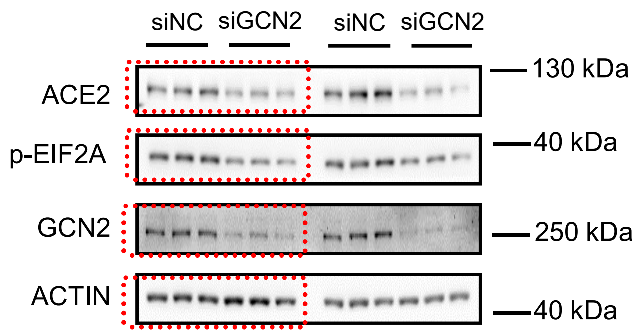

Figure 2d

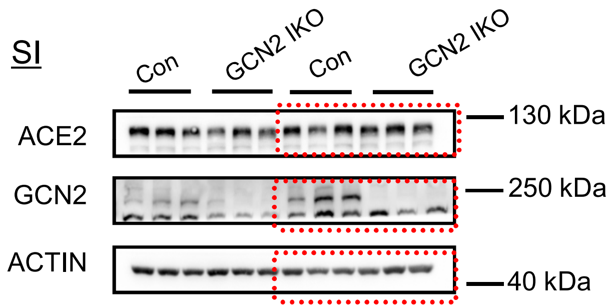

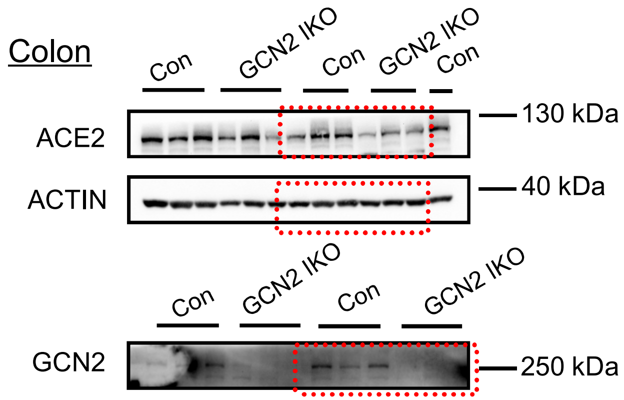

Figure 2e

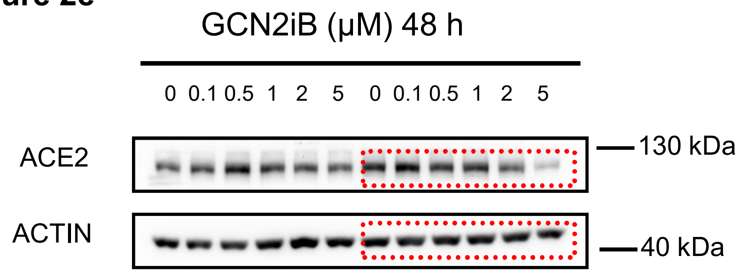

Supplementary Figure 13. Unedited blot for Figure 2b, 2d, and 2e.

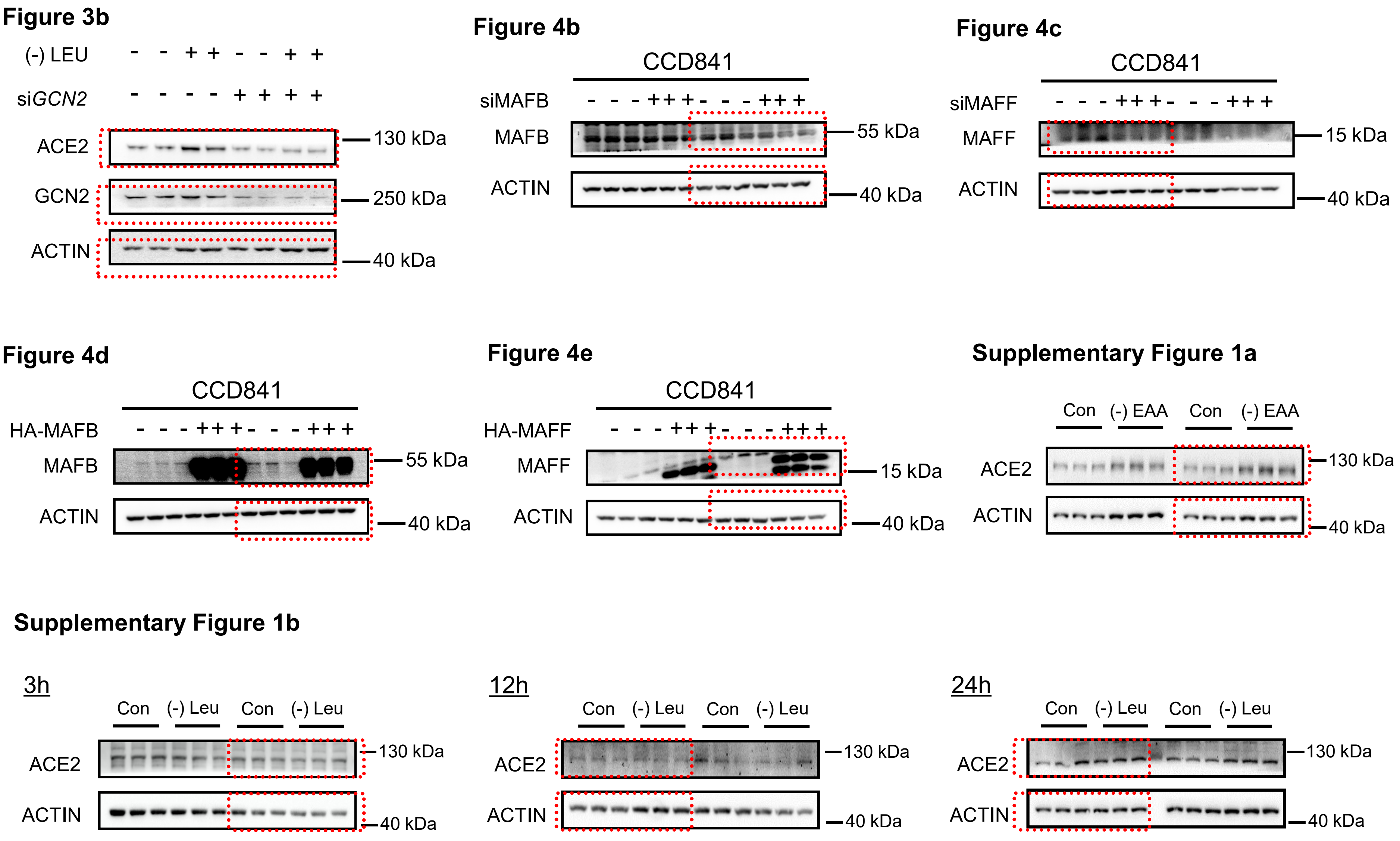

Supplementary Figure 14. Unedited blot for Figure 3b, 4b-4e, Supplementary Figure 1a and 1b.

Supplementary Figure 2

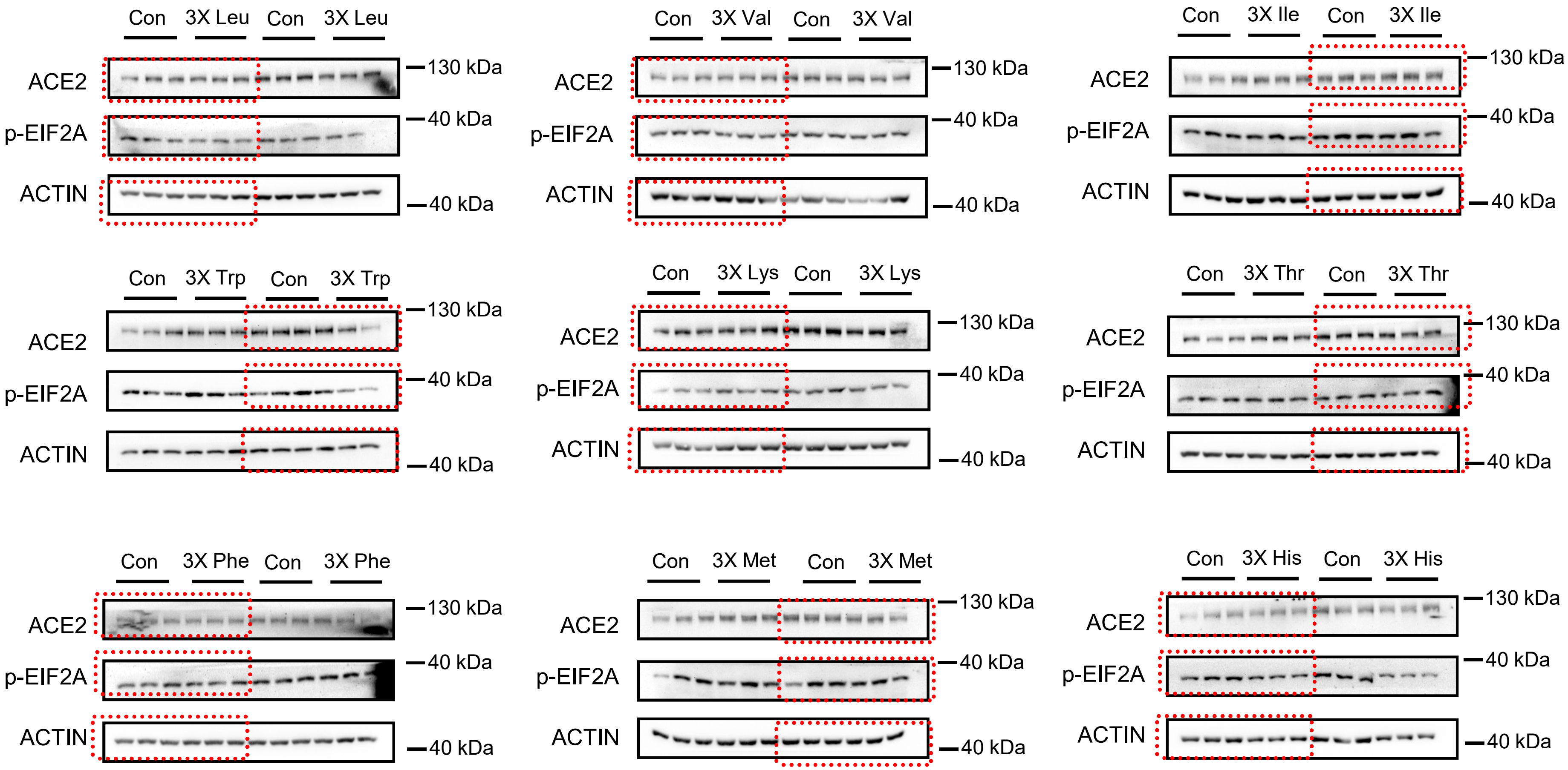

Supplementary Figure 15. Unedited blot for Supplementary Figure 2.

Supplementary Figure 3a

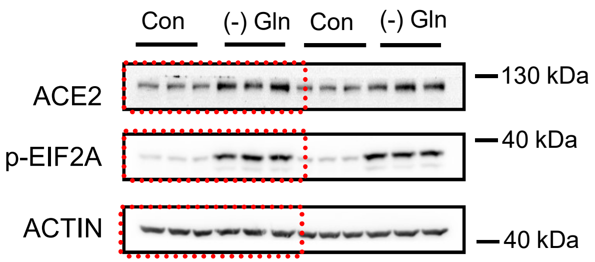

Supplementary Figure 3b

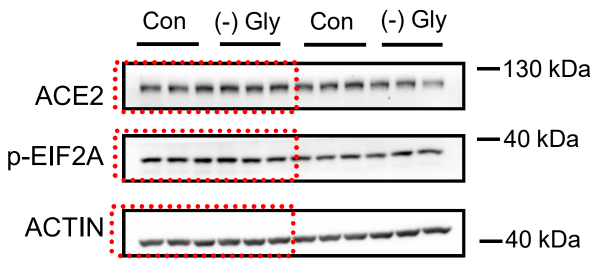

Supplementary Figure 4

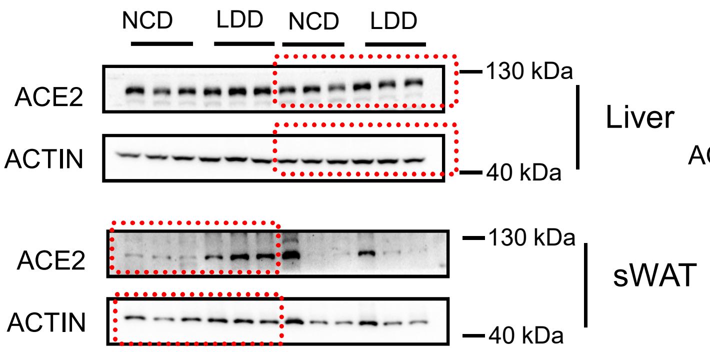

Supplementary Figure 5b

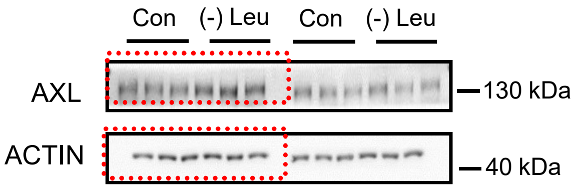

Supplementary Figure 6b

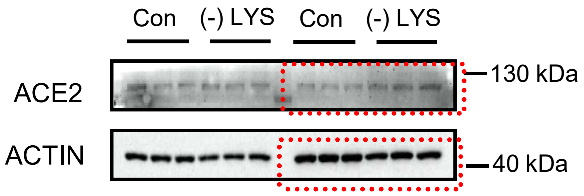

Supplementary Figure 7b

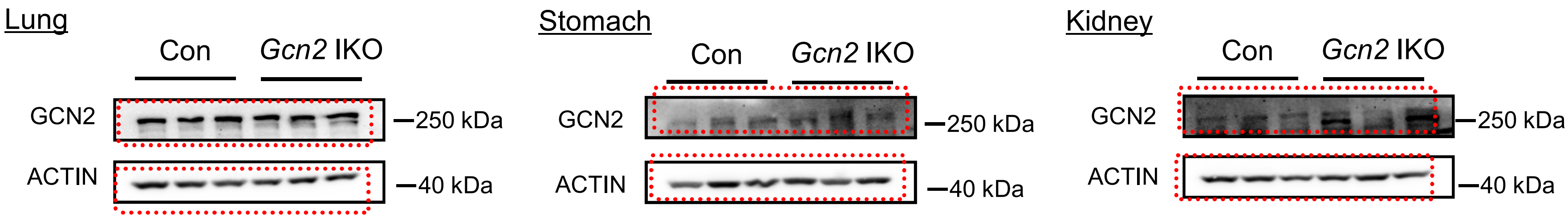

Supplementary Figure 16. Unedited blot for Supplementary Figure 3a, 3b, 4, 5b, 6b, and 7b.

Supplementary Figure 8a

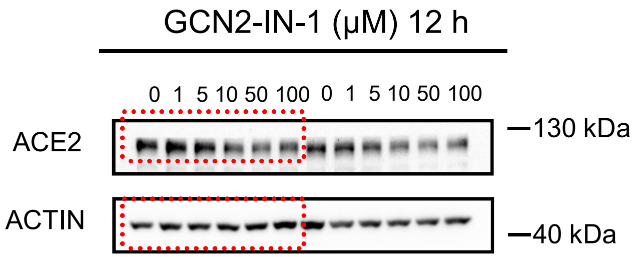

Supplementary Figure 8b

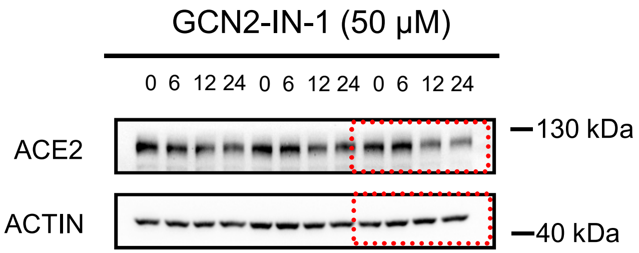

Supplementary Figure 8c

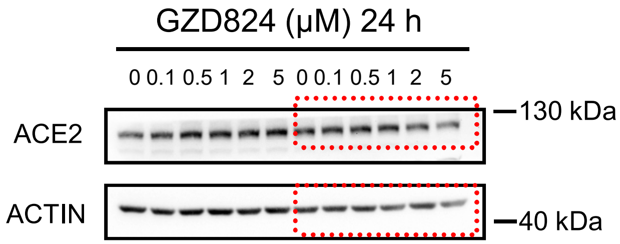

Supplementary Figure 8d

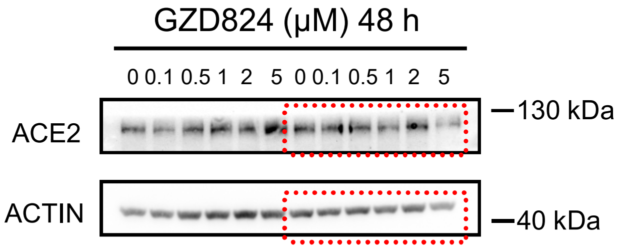

Supplementary Figure 9a

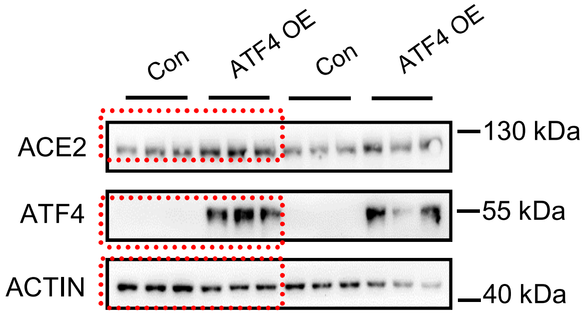

Supplementary Figure 9b

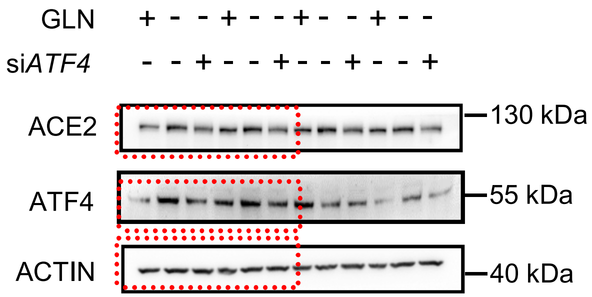

Supplementary Figure 17. Unedited blot for Supplementary Figure 8a-8d, 9a, and 9b.

Genes	Sequence (5’-3’)
JUNB-F	cgGAATTCcgATGTGCACTAAAATGGAACAGCC
JUNB-R	ggGGTACCccTCAGAAGGCGTGTCCCTTGAC
RELB-F	cgGAATTCcgATGCTTCGGTCTGGGCCAGCCTC
RELB-R	ggGGTACCccCTACGTGGCTTCAGGCCCCGG
MAFB-F	cgGAATTCcgATGGCCGCGGAGCTGAGCATG
MAFB-R	ggGGTACCccTCACAGAAAGAACTCGGGAGAGGAG
MAFF-F	cgGAATTCcgATGTCTGTGGATCCCCTATCC
MAFF-R	ggGGTACCccACTTTGGGGAGGGAGCTGAG

Supplementary Table 1. Cloning primers. F: forward primer; R: reverse primer.

Genes	siRNA target sequences (5'-3')
GCN2	CTTGATTATCTGCACAGCAAT
ATF4	GCCTAGGTCTCTTAGATGA
JUNB	GCATCAAAGTGGAGCGCAA
RELB	GAACCATCAGGAAGTAGAC
MAFB	GAGAAACTCGCCAACTCCGGCTTCA
MAFF	CTATCCAGCAAAGCTCTAA
FOSL1	CTAGCACAATTTGCACTAA
FOXP2	GCAAACCAGTGGATTGAAATC
TOX2	CCCAGATCAAGGTGAGACCAA
NC siRNA	TTCTCCGAACGTGTCACGT

Supplementary Table 2. siRNA sequences.

**Components**	**Concentration (mg/L)**
Amino acid	
L-glutamine	584
L-isoleucine	104.4
L-phenylanine	66
L-Arginine HCl	83.84
L-Tyrosine disodium salt dehydrate	103.96
L-Threonine	95
L-Methionine	30
L-Lysine hydrochloride	145.75
L-Histidine	31.03
L-Tryptophan	16
L-Valine	94
L-Serine	42
L-Cystine 2HCl	55.69
Glycine	30
L-Leucine	105
Inorganic Salts	
$CaCl_2$	200
$MgSO_4$*$7H_2O$	200.6
KCl	400
$NaHCO_3$	3700
NaCl	6400
NaH_2PO_4*$2H_2O$	141.3
D-Glucose	4500
Phenol Red	15
Sodium pyruvate	110
Vitamin Solution (100X)	
Choline chloride	100
D-Calcium pantothenate	100
Nicotinamide	100
Folic Acid	100
Pyridoxal hydrochloride	100

Riboflavin	10
Thiamine hydrochloride	100
i-Inositol	200
Sodium Chloride (NaCl)	8500

Supplementary Table 3. Culture medium formulations.

Ingredient (gm）	Control diet	Leu-deficient diet
L-Arginine	10	10
L-Histidine-HCl-H2O	6	6
L-Isoleucine	8	8
L-Leucine	12	0
L-Lysine-HCl	14	14
L-Methionine	6	6
L-Phenylalanine	8	8
L-Threonine	8	8
L-Tryptophan	2	2
L-Valine	8	8
L-Alanine	10	10
L-Asparagine-H2O	5	5
L-Aspartate	10	10
L-Cystine	4	4
L-Glutamic Acid	30	30
L-Glutamine	5	5
Glycine	10	10
L-Proline	5	5
L-Serine	5	5
L-Tyrosine	4	4
Total L-Amino Acids	170	158
Corn Starch	550.5	562.5
Maltodextrin 10	125	125
Cellulose	50	50
Corn Oil	50	50
Hygrogenated Coconut Oil	0	0
Mineral Mix S10001	35	35
Sodium Bicarbonate	7.5	7.5
Vitamin Mix V10001	10	10
Choline Bitrartrate	2	2
Red Dye, FD&C #40	0	0.025
Blue Dye, FD&C #1	0.05	0
Yellow Dye, FD&C #5	0	0.025
Total	1000.05	1000.05

Supplementary Table 4. Diet composition.

Genes	Sequence (5'-3')
mGCN2-F	CCTGCACCATGAGAACATTG
mGCN2-R	CTGCCCAGTTCTTCAGTGT
mACE2-F	TCCAGACTCCGATCATCAAGC
mACE2-R	TGCTCATGGTGTTCAGAATTGT
mGAPDH-F	TGTGTCCGTCGTGGATCTGA
mGAPDH-R	CCTGCTTCACCACCTTCTTGAT
hGCN2-F	TCTATGTAAGCCCAGAGGTCCAA
hGCN2-R	TCCCAGGCTGAAGAGATCCA
hJUNB-F	ACAAACTCCTGAAACCGAGCC
hJUNB-R	CGAGCCCTGACCAGAAAAGTA
hTOX2-F	AGAGCGAGAACAACGAAGACT
hTOX2-R	TGGCCTGATAGGAGTAGGCAG
hRELB-F	CAGCCTCGTGGGGAAAGAC
hRELB-R	GCCCAGGTTGTTAAAACTGTGC
hMAFB-F	TCAAGTTCGACGTGAAGAAGG
hMAFB-R	GTTCATCTGCTGGTAGTTGCT
hFOXP2-F	GCGTCAGGGACTCATCTCC
hFOXP2-R	GAGGTCTAGCCCTCCATGTTTA
hFOSL1-F	CAGGCGGAGACTGACAAACTG
hFOSL1-R	TCCTTCCGGGATTTTGCAGAT
hMAFF-F	CCCCTATCCAGCAAAGCTCTAA
hMAFF-R	GCACCGACAGCCCCATCA
hACE2-F	CAAGAGCAAACGGTTGAACAC
hACE2-R	CCAGAGCCTCTCATTGTAGTCT
hAXL-F	GTGGGCAACCCAGGGAATATC
hAXL-R	GTACTGTCCCGTGTCGGAAAG
hGAPDH-F	CCACCCATGGCAAATTCC
hGAPDH-R	TGGGATTTCCATTGATGACAAG

Supplementary Table 5. qRT-PCR primers. F: forward primer; R: reverse primer; m: mouse; h: human.

Antibodies	Company	Cat No.	Dilution
ACE2	Abclonal	A4612	1:1000
Phospho-EIF2A(Ser51)	Cell Signaling Technology	3398s	1:1000
GCN2	Cell Signaling Technology	65981s	1:1000
AXL	Proteintech	13196-1-AP	1:1000
ATF4	Proteintech	10835-1-AP	1:1000
MAFB	Abclonal	A10077	1:1000
MAFF	Abclonal	A12920	1:1000
β-actin	Proteintech	66009-1-Ig	1:5000

Supplementary Table 6. Antibodies for western blotting.
