# Supplementary material for: Amino acid sensor GCN2 promotes SARS-CoV-2 receptor ACE2 expression in response to amino acid deprivation
Source: Commun Biol. 2022 Jul 1;5:651. doi: 10.1038/s42003-022-03609-0 (PMC9249868; doi:10.1038/s42003-022-03609-0)
Supplement: Supplementary file 5 — Reporting summary [file 42003_2022_3609_MOESM5_ESM.pdf]

nature research

Corresponding author(s): Feifan Guo

Last updated by author(s): May 18, 2022

Reporting Summary

Nature Research wishes to improve the reproducibility of the work that we publish. This form provides structure for consistency and transparency in reporting. For further information on Nature Research policies, see our Editorial Policies and the Editorial Policy Checklist.

Statistics

For all statistical analyses, confirm that the following items are present in the figure legend, table legend, main text, or Methods section.

n/a	Confirmed	
☐	☒	The exact sample size (*n*) for each experimental group/condition, given as a discrete number and unit of measurement
☒	☐	A statement on whether measurements were taken from distinct samples or whether the same sample was measured repeatedly
☐	☒	The statistical test(s) used AND whether they are one- or two-sided<br>*Only common tests should be described solely by name; describe more complex techniques in the Methods section.*
☒	☐	A description of all covariates tested
☒	☐	A description of any assumptions or corrections, such as tests of normality and adjustment for multiple comparisons
☐	☒	A full description of the statistical parameters including central tendency (e.g. means) or other basic estimates (e.g. regression coefficient) AND variation (e.g. standard deviation) or associated estimates of uncertainty (e.g. confidence intervals)
☐	☒	For null hypothesis testing, the test statistic (e.g. *F*, *t*, *r*) with confidence intervals, effect sizes, degrees of freedom and *P* value noted<br>*Give P values as exact values whenever suitable.*
☒	☐	For Bayesian analysis, information on the choice of priors and Markov chain Monte Carlo settings
☒	☐	For hierarchical and complex designs, identification of the appropriate level for tests and full reporting of outcomes
☒	☐	Estimates of effect sizes (e.g. Cohen's *d*, Pearson's *r*), indicating how they were calculated

Our web collection on statistics for biologists contains articles on many of the points above.

Software and code

Policy information about availability of computer code

Data collection	SOFTWARES used for data collection include: Tanon MP for western blot images; ABI 7900 system for RT-PCR□Illumina HiSeq™ 4000 system for microarray.
Data analysis	GraphPad Prism 8 and Microsoft Office Excel were used for statistic analysis; Western Blot images were analyzed using Tanon Gis; Microarray analysis was performed at I-Sanger Cloud Platform (www.i-sanger.com; Shanghai Majorbio, Shanghai, China); No custom code was used.

For manuscripts utilizing custom algorithms or software that are central to the research but not yet described in published literature, software must be made available to editors and reviewers. We strongly encourage code deposition in a community repository (e.g. GitHub). See the Nature Research guidelines for submitting code & software for further information.

Data

Policy information about availability of data

All manuscripts must include a data availability statement. This statement should provide the following information, where applicable:

- Accession codes, unique identifiers, or web links for publicly available datasets
- A list of figures that have associated raw data
- A description of any restrictions on data availability

The microarray datasheet described in the paper has been deposited into Gene Expression Omnibus database (https://www.ncbi.nlm.nih.gov/geo/) with accession number: PRJNA808827. All data presented in this study are available within the Figures and its Supplementary Information file. The source data are available from the corresponding author.

Field-specific reporting

Please select the one below that is the best fit for your research. If you are not sure, read the appropriate sections before making your selection.

☒ Life sciences ☐ Behavioural & social sciences ☐ Ecological, evolutionary & environmental sciences

For a reference copy of the document with all sections, see nature.com/documents/nr-reporting-summary-flat.pdf

Life sciences study design

All studies must disclose on these points even when the disclosure is negative.

Sample size	No sample size calculation was performed, but sample sizes were sufficient to carry out the required experiments with sufficient statistics and standard using t-test for such kind of experiments. To minimize any potential bias, we randomly assigned mice of same genotype to different treatments. In addition, each figure legend describes the number of technical and biological replicates.
Data exclusions	No data were excluded.
Replication	The data reported were generated using at least three different biological replicates in most required experiments. All experimental findings were reproduced for at least twice with similar results.
Randomization	Mice were allocated randomly.
Blinding	Microarray were performed blinded. Other experiments were not blinded, however, we followed standard laboratory procedures of randomization. Each experiment was associated with proper controls, and compared samples were collected and analyzed under the same conditions.

Reporting for specific materials, systems and methods

We require information from authors about some types of materials, experimental systems and methods used in many studies. Here, indicate whether each material, system or method listed is relevant to your study. If you are not sure if a list item applies to your research, read the appropriate section before selecting a response.

Materials & experimental systems

n/a	Involved in the study	
☐	☒	Antibodies
☐	☒	Eukaryotic cell lines
☒	☐	Palaeontology and archaeology
☐	☒	Animals and other organisms
☒	☐	Human research participants
☒	☐	Clinical data
☒	☐	Dual use research of concern

Methods

n/a	Involved in the study	
☒	☐	ChIP-seq
☒	☐	Flow cytometry
☒	☐	MRI-based neuroimaging

Antibodies

Antibodies used

The following primary antibodies were used for Western blotting:
anti-ACE2 (Cat. No. A4612, 1:1000, Abclonal, Wuhan, China);
anti-Phospho-EIF2A(Ser51) (Cat. No. 3398s, 1:1000, Cell Signaling Technology, MA, USA);
anti-GCN2 (Cat. No. 65981s 1:1000, Cell Signaling Technology, MA, USA);
anti-AXL (Cat. No. 13196-1-AP, 1:1000, Proteintech, Chicago, IL, USA);
anti-ATF4 (Cat. No. 10835-1-AP, 1:1000, Proteintech, Chicago, IL, USA);
anti-MAFB (Cat. No. A10077, 1:1000, Abclonal, Wuhan, China);
anti-MAFF (Cat. No. A12920, 1:1000, Abclonal, Wuhan, China);
anti-β-actin (Cat. No. 66009-1-Ig, 1:5000, Proeintech, Chicago, IL, USA).

Validation

Antibodies sourced from commercial corporation are well-validated by the manufacturer and are widely used in the scientific community for Western blotting.
anti-ACE2 (Cat. No. A4612, 1:1000, Abclonal, Wuhan, China) was validated by other users and cited twice;
anti-Phospho-EIF2A(Ser51) (Cat. No. 3398s, 1:1000, Cell Signaling Technology, MA, USA) was validated by other users and cited 561 times;
anti-GCN2 (Cat. No. 65981s 1:1000, Cell Signaling Technology, MA, USA) was validated by other users and cited twice;
anti-AXL (Cat. No. 13196-1-AP, 1:1000, Proteintech, Chicago, IL, USA) was validated by other users and cited twice;
anti-ATF4 (Cat. No. 10835-1-AP, 1:1000, Proteintech, Chicago, IL, USA) was validated by other users and 214 times;
anti-MAFB (Cat. No. A10077, 1:1000, Abclonal, Wuhan, China) with positive WB detected in mouse liver and kidney has been verified

by the company;
anti-MAFF (Cat. No. A12920, 1:1000, Abclonal, Wuhan, China) with positive WB detected in HT-29 cells has been verified by the company;
anti-β-actin (Cat. No. 66009-1-Ig, 1:5000, Proeintech, Chicago, IL, USA) was validated by other users and cited 2678 times.

Eukaryotic cell lines

Policy information about cell lines

Cell line source(s)	CCD841 (ATCC, CRL-1790) and BEAS-2B (ATCC, CRL-9609) cell lines were purchased from Cell Bank of Shanghai Institute of Cell Biology, CAS.
Authentication	The cell lines have been authenticated by Cell Bank of Shanghai Institute of Cell Biology, CAS.
Mycoplasma contamination	The cell culture incubator was treated with mycoplasma scavenger; all cell lines were tested negative for mycoplasma contamination by Cell Bank of Shanghai Institute of Cell Biology, CAS.
Commonly misidentified lines (See ICLAC register)	No commonly misidentified cell lines were used.

Animals and other organisms

Policy information about studies involving animals; ARRIVE guidelines recommended for reporting animal research

Laboratory animals	10-week-old male C57BL/6J wild-type mice were fed a control diet or leucine deprivation diet for 7 days;<br>Gcn2-floxed mice were intercrossed with Villin-cre mice to generate the intestinal epithelial cells-specific Gcn2 IKO mice;<br>10-week-old Gcn2-floxed mice and Gcn2 IKO mice were fed a control diet or leucine deprivation diet for 7 days.<br>All the wild-type mice were obtained from Shanghai Laboratory Animal Co., Ltd. (Shanghai, China); Gcn2-floxed mice and Villin-cre mice were purchased from Shanghai Biomodel Organism Science & Technology Development Co.,Ltd., Shanghai, China.
Wild animals	The study did not involve wild animals.
Field-collected samples	The study did not involve samples collected from the field.
Ethics oversight	The experiments were conducted in accordance with guidelines of the Institutional Animal Care and Use Committee of Fudan University.

Note that full information on the approval of the study protocol must also be provided in the manuscript.
